# The GDPR and the research exemption: considerations on the necessary safeguards for research biobanks

**DOI:** 10.1038/s41431-019-0386-5

**Published:** 2019-04-17

**Authors:** Ciara Staunton, Santa Slokenberga, Deborah Mascalzoni

**Affiliations:** 1 School of Law, Middlesex University, London and Centre for Biomedicine, EURAC, Bolzano, Italy; 2 Faculty of Law, Lund University and Center for Research Ethics and Bioethics Uppsala University Sweden, Uppsala, Sweden; 3 Centre for Biomedicine, EURAC, Bolzano and CRB Uppsala University Sweden, Uppsala, Sweden

**Keywords:** Ethics, Health policy

## Abstract

The General Data Protection Regulation (GDPR) came into force in May 2018. The aspiration of providing for a high level of protection to individuals’ personal data risked placing considerable constraints on scientific research, which was contrary to various research traditions across the EU. Therefore, along with the set of carefully outlined data subjects’ rights, the GDPR provides for a two-level framework to enable derogations from these rights when scientific research is concerned. First, by directly invoking provisions of the GDPR on a condition that safeguards that must include ‘technical and organisational measures’ are in place and second, through the Member State law. Although these derogations are allowed in the name of scientific research, they can simultaneously be challenging in light of the ethical requirements and well-established standards in biobanking that have been set forth in various research-related soft legal tools, international treaties and other legal instruments. In this article, we review such soft legal tools, international treaties and other legal instruments that regulate the use of health research data. We report on the results of this review, and analyse the rights contained within the GDPR and Article 89 of the GDPR vis-à-vis these instruments. These instruments were also reviewed to provide guidance on possible safeguards that should be followed when implementing any derogations. To conclude, we will offer some commentary on limits of the derogations under the GDPR and appropriate safeguards to ensure compliance with standard ethical requirements.

## Introduction

The General Data Protection Regulation (GDPR) seeks to ensure the free movement of data throughout the European Union (EU) and give expression to the right to personal data protection within and beyond the EU, as long as an EU data subject’s data or data collected in the EU are being processed. It details, the lawful basis of the processing of data (Article 6) and delineates prohibitions for processing special categories of data, such as health and genetic data (Article 9), sets out the conditions for consent (Article 7), outlines the individual rights of data subjects (Articles 13–22), and provides data subjects with a mechanism to enforce their rights (Articles 77–84).

The EU aspiration to provide a high level of protection to individuals’ personal data risked placing considerable constraints on scientific research, which was contrary to various research traditions across the EU. Therefore, along with the set of carefully outlined data subjects’ rights, the GDPR also provides for a two-level framework to enable derogations from these rights when scientific research is concerned. First, by directly invoking provisions of the GDPR on a condition that derogations be subject to safeguards that must include ‘technical and organisational measures’, and second, through the Member State law and subject to safeguards.

Although these derogations are allowed in the name of scientific research, they can simultaneously be seen as challenging in light of the ethical requirements and long-standing protection standards for participants in biobanking that have been established in various research-related soft legal tools, international treaties and other legal instruments. In relation to the use of broad consent, the GDPR itself explicitly references such standards and Recital 33 states that it is permitted to give ‘consent to certain areas of scientific research when in keeping with recognised ethical standards for scientific research’. The recognition of ethical standards is here related to broad consent and there is no explicit requirement to consider derogations in line with these ‘recognised ethical standards for scientific research’. Hence, research that could be deemed legal under the GDPR or Member State laws that permits further derogations, might not necessarily be in line with ethical standards that are currently required by research ethics committees (RECs). In other words, a gap between the ethical standard and legal requirements could emerge.

In this article, we review such soft legal tools, international treaties and other legal instruments (collectively referred to here as ‘instruments’) that regulate the use of health research data. We report on the results of this review, and analyse the rights contained within the GDPR and Article 89 of the GDPR vis-à-vis these instruments. These instruments were also reviewed to provide guidance on possible safeguards that should be followed when implementing any derogations. To conclude, we will offer some commentary on limits of the derogations under the GDPR and appropriate safeguards to ensure compliance with standard ethical requirements.

## The ethical concerns related to biobanking

Ethical consideration for biobank-based biomedical research strike a balance between the societal need for scientific development and individual’s dignity and autonomy. The Taipei declaration states that ‘[r]esearch should pursue science advancement and public health development while respecting the dignity, autonomy, privacy and confidentiality of individuals’. Those rights do not include only the direct risks for individuals of being re-identified as ‘the rights to autonomy, privacy and confidentiality also entitle individuals to exercise control over the use of their personal data and biological material’.

In fact concerns about research aims may be unrelated to ‘re-identifiability’ but rather related to possible uses in research, potentially against one person’s ethical beliefs (use of health and genetic data to profile families and new generations, gender profiling, race profiling, communities discrimination, biological weapons based on genetic specificities etc.). Concerns about possible actors involved in the use of data (insurances, private companies etc.) are also major issues as an individual’s willingness to participate in research is often based on trust in specific institutions.

Biobank research is based on long-term organised collections of data and samples that can potentially be used for very diverse research aims. At the time of the collection it is unlikely that the researcher can carefully and truly inform participants on all possible future research uses, but it is possible to provide good information on the governance of data. Recent instruments show that consent (previously the main legal basis in which to lawfully conduct research) has been in fact paired with strong governance measures and third party oversight mechanisms to be adopted by institutions to regulate sharing, accessing and use of the data. Making public governance procedures on decision making is part of the trustworthiness of an institution and has ‘replaced’ in some cases specific consent as a hardly implementable option [1, 2]. Strong governance and ethical oversight has been proposed to support the oversight of waivers of consent in the biobank practice [3] along with forms of ongoing dynamic options [4]. Detailed governance information comprising oversight policies are usually available to participants at the time of consent.

Processing of data including secondary uses is a very sensitive area in research, not only from a legal point of view but also from an ethical one. Those concerns lead to a great emphasis on principles of transparency, trust and partnership and to the development of models for ongoing information and dynamic consent accounting for changing landscapes of uses including secondary findings [5].

The GDPR provisions on research are built on exceptions and national derogations to a law that otherwise is committed to paying great attention to human rights. However, it is unclear whether those provisions are balanced by appropriate safeguards or if they are challenging recent advancement in ethics, leading one to question whether a project that is legally compliant under the GDPR is in fact ethically compliant.

## The ethical rip-off: GDPR perspectives on data subject and biobanking

### Lawful processing of data under the GDPR

Article 6 GDPR sets forth legality requirements, but these requirements must be viewed jointly with the special protection afforded to special categories of data, including health and genetic data under Article 9 GDPR. Generally for biobanking purposes, the following three lawfulness basis are of particular relevance. First, the data subject’s consent under Article 6 (1)(a). Second, the performance of a task carried out in the public interest under Article 6(1)(e). Third, processing is necessary for the purposes of the legitimate interests pursued by the controller or by a third party under Article 6(1)(f), except where such interests are overridden by the interests or fundamental rights and freedoms of the data subject, which require protection of personal data, in particular where the data subject is a child.

A distinction can be drawn between primary research and secondary biomedical research based on data and samples. While lawful basis of primary research could be consent based, which might not necessarily be so for secondary use of personal data or research using residual biological material. In the latter cases, the claim of legitimate interest is of particular importance. When data are not processed based on the individual’s consent, the requirements set in Article 6(4) shall be met, which includes the existence of appropriate safeguards.

Article 9(1) prohibits the processing of special information that includes genetic information, but Article 9(2)(j) allows for processing the genetic data as part of a special category of data if ‘processing is necessary for archiving purposes in the public interest, scientific or historical research purposes or statistical purposes.’ Furthermore, additional measures could be taken under Article 9(4) GDPR, through which Article 9(2) GDPR scope may be implicitly expanded allowing further conditions for processing health and genetic data, or constraining it. When applying Article 9(2)(j), this processing must be ‘in accordance with Article 89(1) based on Union or Member State law, which shall be proportionate to the aim pursued, respect the essence of the right to data protection and provide for suitable and specific measures to safeguard the fundamental rights and the interests of the data subject.’ In other words, for example, a Member State may attribute particular value to biobank research; they may limit a data subjects right to control the use of their data in research by removing the consent requirement for the processing of genetic data in biobanking, provided the national law respects the principle of proportionality, the essence of the right of data protection, and provides for suitable and specific measures to safeguard the rights and interests of data subjects. Yet, as further below is discussed, the GDPR is not very informative on these measures.

### Individual rights and research-related derogations under GDPR

The GDPR provides data subjects with a number of rights. In biobanking, the following are of key importance: right to information (in particular, Art. 12–14), access rights (Art. 15), right to rectification (Art. 16), right to erasure (Art. 17), right to restriction of processing (Art. 18), right to data portability (Art. 20), right to object (Art. 21). Additional protective measures include, for example, a notification entitlement, providing the data subject has triggered it (Art. 19). Further to these rights and protection measures, at the data subject’s disposal is access to justice, including remedies, liability and penalties (Art. 77–84). However, this set of rights does not seem to be at the data subject’s disposal in all cases in biobank research. The exact set of rights at the data subject’s disposal depends on several circumstances, key being whether the Member State has derogated from the GDPR provisions under Article 89.2 GDPR, and/or the biobank relies on derogations through directly invoking the GDPR provisions under Article 89(1) GDPR and subsequent provisions throughout the GDPR.

Article 89(1) GDPR enables processing of genetic data for scientific research purposes, if there are appropriate safeguards for the rights and freedoms of the data subject. While the GDPR does not exhaustively specify what those safeguards are, it indicates their purpose is to ‘ensure that technical and organisational measures are in place in particular in order to ensure respect for the principle of data minimisation.’ These measures may include pseudonymisation provided it enables meeting the intended research purposes. In situations where data are not collected directly from the subject (e.g., residual material use), data subject’s right to information under Article 14 could be derogated from. Other rights that can be derogated from include the right to erasure under Article 17, as well as the right to object under Article 21.

In addition to these rights, EU or Member State law may provide for derogations from a set of other rights, provided that appropriate safeguards for the rights and freedoms of the data subject are in place. These derogations relate to the data subject’s access rights (Art. 15), right to rectification (Art. 16), right to restriction of processing (Art. 18), as well as right to object (Art. 21), and as specified in Article 89(2) GDPR, they can be applied if the derogations are necessary for research purposes.

While the data subject could remain with the data portability rights and additional protective measures, namely, the notification entitlement, these measures have a rather limited scope. First, the notification entitlement is closely related to information. If the data subject’s right to information is waived, the notification entitlement under Article 19 could be affected. Data portability, however, has a rather limited scope and it is inapplicable if biobank research relates to the performance of a task carried out in the public interest, as stated in Article 20 [6].

The potential scope of the research exemptions by directly invoking the GDPR and through the Member State laws that enables further derogations is so wide that, if applied to its full extent, not only is a data subject’s consent not necessary for the processing of personal data for research, but also the data subject can be stripped from a number of rights and others can be rendered ineffective, leaving the data subject with an enforcement mechanism only. This means, if, for example, data subjects do become aware of processing their data for biobank research, they might have no right to access information on this research or object to the research. The data subject could have no right to restrict the use of their data for research, correct any errors or request to erase the data. They would, however, be able to lodge a complaint with the data protection authority, and thus could retain some oversight [7]. However, one can then question the effectiveness of such a mechanism from a data subject’s perspective if there are no rights on the part of data subject what to oversee. The research exemption could thus undo many of the stated aims of the GDPR for research, and put at stake its own objective—the protection of privacy.

## Methods

Soft legal tools, international treaties and other legal instruments (hereinafter collectively called ‘instruments’) that are influential on the regulation of health research were identified. Our review was limited to instruments that may be relevant to some or all Member States of the EU, which are either directly applicable to the Member States or to certain professions within the Member States. Our review did not include international consortia guidelines. A codebook was developed by CS and DM. All instruments were imported into Nvivo 11 and coded using thematic content analysis. A summary of the reviewed legally binding instruments can be found in Table 1, instruments binding on particular bodies can be found in Table 2 and legally persuasive instruments can be found in Table 3.

**Table 1:** Instruments reviewed

	Instrument	Year	Publishing body	Applicable to
1.	GDPR	2016	European Union	Any company/entity, which processes personal data as part of the activities of one of its branches established in the EU, regardless of where the data are processed; or a company established outside the EU offering goods/services or monitoring the behaviour of individuals in the EU
2.	Charter of Fundamental Rights and Freedoms of the European Union	2000	European Union	Member States of the EU in so far as they are implementing/applying EU law
3.	Council of Europe	1953	European Convention on Human Rights	Member States of the Council of Europe and Members States of the EU
4.	Council of Europe	1997	Convention for the Protection of Human Rights and Dignity of the Human Being with regard to the Application of Biology and Medicine: Convention on Human Rights and Biomedicine (Oviedo Convention)	Twenty-nine states that have signed and ratified the Convention (a further five have signed but not ratified)
5.	Council of Europe	1980 (revised 2018)	Convention for the protection of individuals with regard to the processing of personal data	States that have signed and ratified the Convention
6.	United Nations	1966	International Convention on Economic, Social and Cultural Rights	Countries that have ratified the Convention

**Table 2:** Instruments binding on particular bodies

	Instrument	Year	Publishing body	Applicable to
1.	Declaration of Helsinki	The 2013 edition	World Medical Association	Doctors (but persuasive for all health researchers)
2.	Declaration of Taipei on Ethical Considerations Regarding Health Databases and Biobanks	2002 (revised 2016)	World Medical Association	Doctors (but persuasive for all health researchers)

**Table 3:** Legally persuasive instruments

	Instrument	Year	Publishing body	Applicable to
1.	Recommendation CM/Rec(2016)6 of the Committee of Ministers to member States on research on biological materials of human origin	2016	Council of Europe	47 Member States of the Council of Europe
2.	Recommendation on Health Data Governance	2017	OECD	35 Member States
3.	OECD Guidelines on Human Biobanks and Genetic Research Databases	2009	OECD	35 Member States
4.	Principles and Guidelines for Access to Research Data from Public Funding	2007	OECD	35 Member States
5.	Universal Declaration on Human Rights	1948	UN	
6.	Universal Declaration on Bioethics and Human Rights	2005	UNESCO	UN Members
7.	International Declaration on Human Genetic Data	2003	UNESCO	UN Members
8.	Universal Declaration on the Human Genome and Human Rights	1997	UNESCO	UN Members
9.	International Ethical Guidelines for Health-related Research Involving Humans	2016	Council for International Organizations of Medical Sciences	Health-related researchers

## Results

While the GDPR grants a number of rights to the data subjects (and simultaneously takes them away for research purposes), the focus of the other instruments is on the safeguards that must be in place for research participant data. Two exceptions are the right to information and the right to access.

### Right to information

The requirements around information in the reviewed instruments is generally bound up with the consent requirements. All instruments, with the exception of the 2007 OECD Guidelines, have requirements regarding consent. It is clear from the instruments reviewed that the preference is in favour of specific, informed and written consent, where possible. Information is requested in all guidelines and should be given in plain language.

All legally binding instruments refer to the importance of informed consent, but do not necessarily set it as an obligatory pre-condition for secondary research. In addition, the GDPR introduces the possibility for electronic online consent as a viable option, provided the consent is clear and concise (Recital 32). Under the GDPR, a data subject must be informed about (among others) the identity and contact details of the data controller, the data protection officer, the purposes for which the data will be processed, the recipients of the data, the duration of storage and the right to withdraw consent if consent is the lawful basis of processing (Article 13). The 2016 Council of Europe Recommendation requires that participants be informed of the conditions applicable to the storage of the materials, including access and possible transfer policies and any relevant conditions governing the use of the materials, including re-contact and feedback (Article 10). The Council of Europe 2018 Convention mandates data subjects to be told the legal basis and the purposes of the intended processing, the categories of personal data processed, the recipients or categories of recipients of the personal data, and their rights as a data subject (Article 8).

Eminent guidance comes from the World Medical Association (WMA), historically among the first institutions to convey ethical rules for research. Its instruments sets professional standards for doctors, and are of importance in the regulation of health research globally. The Declaration of Helsinki requires that participants be informed of the aims of the research, methods, sources of funding, any possible conflict of interests, benefits and risks, institutional affiliations of the researchers and any other relevant information (Principle 26). The WMA Taipei Declaration seeks to regulate health databases and biobanks and provides more details on the requirements for consent: participants have to be informed about the purpose, the risks and burdens, storage and use of data and material, the nature of the data or material to be collected, the procedures for return of results including incidental findings, the rules of access to the health database or biobank, the protection of privacy, the governance arrangements, procedures to inform participants about the impact of anonymisation of data, their fundamental rights and safeguards as established in the Declaration, and when applicable, commercial use and benefit sharing, intellectual property issues and the transfer of data or material to other institutions or third countries (Article 12).

Allied with this extensive right to information, are the provisions on the right to withdraw consent and the obligation to inform data subjects about this right. Article 7(3) of the GDPR states that data subjects can withdraw their consent at any time and that it ‘shall be as easy to withdraw as to give consent’. The UNESCO Declaration on Bioethics (Article 6(1)), the Taipei Declaration (Article 15) and the 2017 OECD Recommendation (Article 5(2)) states that there must be procedures in place to accommodate withdrawal of consent. CIOMS states that participants need to be informed of their right to withdraw their consent, procedures need to be put in place, any withdrawal should be formalised and written, and future use of data are not permitted after this withdrawal.

Most instruments do recognise the limits on the withdrawal of consent. The 2009 OECD Guideline states that at the time of consent participants must be informed about the limits of a withdrawal of consent (Principle 4.G) as does the 2016 Council of Europe Recommendation (Article 13). Specifically, this can only be done for identified genetic data (UNESCO Declaration on Genetic Data, 2016 CoE Recommendation). It is not clear whether these limitations go beyond the practical limitation of the non-removal of anonymous data. Withdrawal of consent is thus seen of importance in both legally binding and other instruments where the legal processing of data are based on consent, but the limits on this withdrawal is recognised. Instruments that are of persuasive value state that the limits on the withdrawal of consent should be communicated to the participants.

### Right to access

The GDPR, the Council of Europe 2018 Recommendation, the Taipei Declaration, the Oviedo Convention, the UNESCO Declaration on Genetic Data and the Council of Europe Recommendation all discuss the right of an individual to access their data, but there subtle differences. Under the GDPR, the data subject has the right to access information about their personal data including confirmation as to whether a data controller is processing their personal data and the purpose, other recipients of their personal data including to third countries (and the safeguards in place), where the data controller obtained the data when the data were not collected from the data subject, and expected storage period or the criteria to determine the storage period (Article 15). Article 15(3) also gives the data subject the right to access a copy of their personal data that is being processed. The Oviedo Convention states that individuals are ‘entitled to know any information collected about his or her health’ (Article 10(2)).

The WMA Taipei Declaration provides that individuals have the right to request and be provided with information about their data and requires health databases to adopt measures so that they can inform individuals about their activities (Article 14).

The 2017 OECD Guideline states that individuals must be provided with ‘information about the processing of their personal health data, including possible lawful access by third parties, the underlying objectives behind the processing, the benefits of the processing’. The Council of Europe also specifies that the management and use of the data should be made available to ‘persons’ concerned’, but this refers to the data collection in general and not the individual data (Article 6(7)). The 2006 UNESCO Declaration states that no one should be denied access to their data ‘unless domestic law limits such access in the interest of public health, public order or national security’ (Article 13).

There are limitations on this right to access. The GDPR states that the right to access does not apply when the data are anonymous and the right to access does fall within one of the possible national derogations under Article 89(2). The Oviedo Convention simply states that the right to access may be restricted in ‘exceptional’ cases with no further elaboration. The UNESCO Declaration on Genetic Data also states that the right to access does not apply when the data are anonymous and that the right to access can be limited by law in ‘the interest of public health, public order or national security’ (Article 13).

Some instruments also touch on feedback to participants as distinct from access to data. Generally, where it is discussed, a policy on feedback is required, but not necessarily that feedback must take place. Discussion on feedback of findings is confined to the non-binding instruments. The 2009 OCED Guidelines discusses ‘feedback’ to participants. It does not mandate that there is feedback to participants, only that there is a policy in place (Principle 4.9) and the results that can be feedback (Principle 4.14). The annotations to the Guideline do state that participants should be provided with information about the type of research that may be carried out on the data, whether it will be used for commercial research and if it will be transferred abroad. The 2016 CoE Recommendation similarly states that there must be a policy in place on feedback of findings and the importance of counselling (Article 17).

### Right to rectification and right to erasure

The GDPR is alone in discussing the right to rectification and the right to erasure. None of the other instruments reviewed discussed these rights. The right to erasure may be linked to a right to withdraw from research as the withdrawal from any study may include the erasure of a participants’ data, but any recognised right to erasure would go further than a right to withdrawal. The right to withdrawal is arguably limited to ongoing and future research, whereas a right to erasure would include the removal from future, ongoing and past research, including potentially published research.

### The right to data portability and the right to object

Article 20 of the GDPR gives data subjects the right to move their data from one data controller to another. The 2017 OECD Recommendation is the only other regulation reviewed that provides that data subjects should be permitted to request the sharing of their data for health-related purposes, and if this is rejected, they must be provided with a legal basis for that decision (Section 5(ii)(a) and (b)).

Article 21 of the GDPR provides data subjects with the right to object to the processing of their personal data, including for research purposes. Once again, the 2017 OECD Recommendation is the only regulation reviewed that states that where consent is not the lawful basis of processing, individuals should be able to object to the processing of their personal information. If this cannot be honoured, they should be provided with a relevant legal basis for the decision (Section 5(ii) (a) and (b)).

### Safeguards to protect data subjects

Article 89(1) of the GDPR states that safeguards ‘shall ensure that technical and organisational measures are in place in particular in order to ensure respect for the principle of data minimisation’. These measures ‘may include pseudonymisation’, but offer no further insight into what they may also be.

Looking at the instruments reviewed, the importance of clear governance procedures (and by implication, transparency) is essential in the oversight of the use and re-use of data. This is of particular importance when the data subject has not provided specific consent to the use of the data. This may be in a manner prescribed by law, a requirement of institutional oversight that may include approval by an ethics committee or some other body, requirement of safeguards, or a combination. As outlined in Table 4, there are three levels of oversight or protections discussed in the instruments: a legislative framework, institutional oversight that includes independent ethical review and provides for other safeguards.

**Table 4:** Governance required for secondary use

Legislative framework	Institutional oversight	Subject to safeguards
Council of Europe 2016 recommendation; Universal Declaration on the Human Genome and Human Rights; OECD 2007 Principles; UNESCO Declaration on Genetic Data	Council of Europe 2016 recommendation; Oviedo Convention; CIOMS Guidelines; Declaration of Helsinki; Taipei Declaration; UNESCO Declaration on Genetic Data	GDPR; OECD 2017 recommendation

## Discussion

The instruments reviewed have a different aim than the GDPR [8], are specifically developed for research and they do provide additional guidance that should be followed to ensure that biobanks meet standard ethical practice. First, although the derogations under the GDPR are potentially quite wide ranging, the instruments reviewed put some limits on these derogations, specifically in relation to the right to information and the right to access. Regarding the right to information, data subjects should be informed about re-contact, feedback, storage, and withdrawal of consent and any possible limits. Similarly, the instruments reviewed do strongly suggest that data subjects should be able to access their personal data, but a distinction is drawn between access of data and feedback of findings/results. The instruments do not mandate feedback of findings, but rather state that a policy should be in place. Whether there is in practice any real distinction between access of genetic data and feedback of findings, it is clear that at a minimum, biobanks should have a policy on feedback of findings in place stating whether or not this is foreseen.

Second, the instruments do provide guidance on possible safeguards. Article 89(1) and Article 89(2) speaks of the importance of the rights and freedoms of the data subject’ and this should be considered when invoking the research exemption. However, there is little guidance within the GDPR itself on striking the balance between research and individual rights. The words ‘in particular’ and ‘may include’ in Article 89(1) of the GDPR indicates that the safeguards include but are not confined to technical and organisational measures and pseudonymisation. The Article 29 WP (now the European Data Protection Board) states that safeguards could include ‘Information Security Management Systems (e.g., ISO/IEC standards) based on the analysis of information resources and underlying threats, measures for cryptographic protection during storage and transfer of sensitive data, requirements for authentication and authorisation, physical and logical access to data, access logging and others’ [9]. However, data protection is much more than a technical issue requiring technical solutions and the Article 29 WP has also spoken of the need for ‘additional legal, organisational and technical safeguards’ [9]. The safeguards must respond to the multitude of legal, ethical and social risks that are associated with the sharing of data. These risks are not static and change over time. Thus, any safeguards must be dynamic and responsive to an evolving science. In determining the safeguards that should be adopted, this review makes it clear that any derogations are subject to two pertinent factors.

First the importance of clear and transparent policies on a multitude of issues is evident. These policies may include policies on data transfer, feedback of findings, storage of data, withdrawal of consent, re-contact of data subjects, access requests from third parties, access requests of data subjects, governance, and (where applicable) intellectual property and commercial use. It is essential that biobanks have these policies in place and that they are publicly available. Having these policies available at the outset is also in line with the GDPR’s policy of privacy by design and default.

Second, it is evident that clear and transparent governance procedures that oversee the use of data are essential in protecting the rights of the data subject. Once again this is in line with the GDPR and the importance of transparency. A coherent and robust governance structure is key in fostering trust and trustworthiness that is so important in biobanking [10]. What emerges from the instruments reviewed is that there are broadly three levels to a governance structure that Member States should follow: a national legal and ethical framework; independent and interdisciplinary review and oversight of the research; and local policies on data sharing and protection.

At a minimum, national legislation should provide for the legal basis of processing of personal data for research purposes. The legislation should also mandate for local, independent approval and oversight prior to the use of data in research. Generally this will be in the form of institutional research ethics review. However, while each research project that seeks to collect and use personal data are currently subject to independent ethics review, the subsequent use and access of this data may not, with no body ensuring that the rights of data subjects are protected. Controlling access to the subsequent use of data are essential to ensure that there are no undue risks to the participant, and this review points to the importance of independent review of access requests for data. An independent body is best placed and likely to have the necessary expertise to consider the potential heterogeneous risks of an access request and these risks should not outweigh the benefits [11].

Third, each biobank must have transparent policies in place regarding the use of personal data. They should include but are not limited to policies on access, information, use and re-use of data, transfer to third parties and feedback of findings. These policies must be made publically available and submitted to a local ethics committee as part of the research protocol.

Finally, members of an ethics committee may be faced with a situation whereby a study under review meets the requirements and derogations under the GDPR, but have ethical concerns about the research. A resolution to this gap between the law and ethics may in part depended on the national legal order that is in place, but the purpose of such committees is to ensure the ethical conduct of research. The law is a minimal standard to which researchers must comply with and the Article 29 Working Party Guidelines on consent state that (where consent is the legal basis for processing) ‘consent for the use of personal data should be distinguished from other consent requirements that serve as an ethical standard or procedural obligation’ [12]. Ethics committees are not necessarily under the obligation to approve research that meets this legal threshold, but fails to meet ethical criteria.

## Conclusion

The GDPR provides for derogations on certain individual rights for research on two separate grounds, but they must be subject to safeguards and provided for by Member State law. There is little insight or guidance contained within the GDPR as to the appropriate safeguards that must be in place, which is alarming considering the potential scope of the derogations. This review makes it clear that a full implementation of the derogations as provided for under the GDPR may render the research unethical and not in line with individuals interests. These instruments also suggest that clear governance procedures and policies on the use and re-use of personal data can go some way towards ensuring that there are necessary safeguards in place to ensure the protection of personal data. This would also ensure that any derogations continue to be in line with the GDPR’s transparency requirements and privacy by design and default. By following these necessary safeguards, biobanks can ensure that they may continue to conduct research, which ensuring the protection of personal data. In this way, research will not trump data protection, but there will be a balance of the (at times) two competing interests [13].

## References

[1] Mascalzoni D, Dove ES, Rubinstein Y, Dawkins H, Kole A, McCormack P (2015). International Charter of principles for sharing bio-specimens and data. Eur J Hum Genet.

[2] Boers S, van Delden J, Bredenoord A (2015). Broad consent is consent for governance. Am J Bioethics.

[3] Gainotti S, Turner C, Woods S, Kole A, McCormack P, Lochmuller H (2016). Improving the informed consent process in international collaborative rare disease research: effective consent for effective research. Eur J Hum Genet.

[4] Kaye J, Whitley EA, Lund D, Morrison M, Teare H, Melham K (2015). Dynamic consent: a patient interface for twenty-first century research networks. Eur J Hum Genet.

[5] Budin-Ljøsne I, Teare H, Kaye J, Beck S, Beate Bentzen H, Caenazzo (2017). Dynamic consent: a potential solution to some of the challenges of modern biomedical research. BMC Medical Ethics.

[6] Chassang G, Southerington T, Tzortzatou O, Boeckhout M, Slokenberga S (2018). Data portability in health research and biobanking**:** legal benchmarks for appropriate implementation. Eur Data Protection Law Rev.

[7] Slokenberga Santa, Reichel Jane, Niringiye Rachel, Croxton Talishiea, Swanepoel Carmen, Okal June (2018). EU data transfer rules and African legal realities: is data exchange for biobank research realistic?. International Data Privacy Law.

[8] Slokenberga S (2018). Biobanking between the EU and third countries — can data sharing be facilitated via soft regulatory tools?. Eur J Health Law.

[9] Article 29 Data Protection Working Party. Advice paper on special categories of data (“sensitive data”). Ares (2011) 444105.

[10] Whitley EA, Kanellopoulou N, Kaye J (2012). Consent and research governance in Biobanks: evidence from focus-groups with medical researchers. Public Health Genomics.

[11] Shabani M, Borry P (2018). Rules for processing genetic data for research purposes in view of the new EU General Data Protection Regulation. Eur J Hum Genet.

[12] Article 29 Data Protection Working Party. Guidelines on consent underRegulation 2016/679. WP259 rev.01.

[13] Mascalzoni D, Beate Bentzen H, Budin-Ljøsne I, Bygrave LA, Bell J, Dove ES (2019). Are requirements to deposit data in research repositories compatible with the European Union’s general data protection regulation?. Ann Intern Med.

